# Editorial: Rare dyslipidemias

**DOI:** 10.3389/fgene.2023.1248435

**Published:** 2023-07-17

**Authors:** Fouzia Sadiq, Robert A. Hegele, Alberico L. Catapano, Urh Groselj

**Affiliations:** ^1^ Shifa Tameer-e-Millat University, Islamabad, Pakistan; ^2^ Department of Medicine, Western University, London, ON, Canada; ^3^ Department of Pharmacological and Biomolecular Sciences, University of Milano, Milan, Italy; ^4^ IRCCS Multimedica, Milano, Italy; ^5^ Department of Pediatric Endocrinology, Diabetes and Metabolism, University Medical Center, University Children’s Hospital, Ljubljana, Slovenia; ^6^ University of Ljubljana, Faculty of Medicine, Ljubljana, Slovenia

**Keywords:** rare diseases, familial hypercholesterolemia, dyslipidemia, atherosclerosis, cardiovascular disease

Rare diseases are defined in the European Union (EU) as those that affect less than 1 person in 2000; in total, between 6,000 and 8,000 different rare diseases affect an estimated 30 million people in the EU (European Commission, 2021). Rare dyslipidemias encompass a diverse group of rare inherited metabolic disorders that are either autosomal dominant, codominant, semi-dominant or recessive (Hegele et al., 2020). At least 25 different monogenic rare dyslipidemias have been identified, caused by pathogenic variants in 23 genes with varied biochemical and clinical features (Hegele et al., 2015). Owing to their complex etiology and clinical features, these diseases pose a significant challenge in diagnosis, which is generally based on the analysis of clinical phenotypes; however, genetic testing provides a definitive diagnosis (Hegele et al., 2020). Extreme deviations in lipoprotein levels particularly at a younger age and with a positive family history of the disease raise suspicion for these rare disorders (Berberich and Hegele, 2022).

Rare dyslipidemias are characterized by abnormal levels of total and low-density lipoprotein (LDL) cholesterol, triglycerides (TG), lipoprotein (a) [Lp(a)], and high-density lipoprotein (HDL) cholesterol. These disorders can have long-term life-threatening consequences including increased risk for atherosclerotic cardiovascular disease (ASCVD), which is the leading cause of morbidity and mortality around the world. Other complications include pancreatitis, fatty liver disease, and fat-soluble vitamin deficiencies (Berberich and Hegele, 2022).

The Research Topic collection on “*Rare dyslipidemias*” presents papers on various aspects of several related disorders, including homozygous familial hypercholesterolemia, familial chylomicronemia syndrome due to different genetic causes, hypobetalipoproteinemia, hypoalphalipoproteinemia, dysbetalipoproteinemia, cerebrotendinous xanthomatosis, and lysosomal acid lipase deficiency.

Homozygous familial hypercholesterolemia (HoFH), marked by exceedingly high levels of LDL cholesterol (>400 mg/dL or >10 mmol/L) due to an impaired clearance of LDL particles, is a major rare dyslipidemia with estimated prevalence of 1 in 300,000 individuals (Berberich and Hegele, 2022; Tromp et al., 2022; Cuchel et al., 2023). It is caused by bi-allelic pathogenic variants in the *LDLR* gene encoding LDL receptor in 85%–90% of cases, in the *APOB* gene encoding apolipoprotein (apo) B in 5%–10% of cases and in the *PCSK9* gene encoding proprotein convertase subtilisin/kexin type 9 in 1%–3% of cases. Clinical features include tendon xanthomas, corneal arcus, and very premature ASCVD and aortic disease (Cuchel et al., 2023; Hegele et al., 2020). Globally, <10% of the FH population is diagnosed and treated, due to a lack of awareness among the general public and medical community (Vallejo-Vaz et al., 2021; Wilemon et al., 2020; Sadiq et al., 2023). Effective management of HoFH remains challenging and includes lipoprotein apheresis to delay ASCVD (Cuchel et al., 2023). Kayikcioglu et al. present an interesting report on the use of low-dose lomitapide on top of standard lipid-lowering therapy resulting in decreased apheresis frequency.

Familial chylomicronemia syndrome (FCS), is a rare (1 in 1,000,000) recessive disorder, that results from the decreased clearance of large TG-rich lipoproteins (chylomicrons), even after prolonged fasting. The major life-threatening complication of FCS is acute pancreatitis, specifically at TG > 10 mmol/L (>885 mg/dL) (D’Erasmo et al., 2019). Most FCS cases are caused by biallelic pathogenic variants in the *LPL* gene encoding lipoprotein lipase (LPL), the enzyme responsible for the breakdown of TG within chylomicrons and very-low-density lipoproteins (VLDL) (Hegele et al., 2020). Causal variants in other genes such as *APOC2* encoding apo C-II, *APOA5* encoding apo A-V, *LMF1* encoding lipase maturation factor 1, *GPIHBP1* encoding glycosylphosphatidylinositol-anchored HDL binding protein 1 account for 10%–20% of the FCS cases (D’Erasmo et al., 2021). A systematic review by Sustar et al. covers the spectrum of *GPIHBP1* gene variants among the hypertriglyceridemia population. Whole genome sequencing of a Chinese proband followed by functional analyses of the genes revealed the digenic origin of hypertriglyceridemia caused by *LMF1* and *LPL* gene double heterozygosity in the patient (Guo et al.). Another study from China reported variants in the *GPD1* gene encoding glycerol-3-phopshate dehydrogenase 1 and their effect on transient infantile hypertriglyceridemia, a rare autosomal recessive disorder that may increase the risk of cardiovascular and metabolic disorders later in life (Wang et al.).

Consanguineous marriages are still prevalent in South Asia, the Middle East, and North Africa for local cultural and social reasons, and are associated with increased risk for inherited diseases with severe health consequences. Al-Waili et al. reported a novel pathogenic variant in the *LPL* gene in a family with double-cousin marriages. In addition, digenic (double heterozygous) pathogenic variants in *LPL* and *APOA5* genes were identified in another Omani family. Similarly, Ayoub et al. reported the first case with pathogenic variants of both *LPL* and *PCSK9* genes in a Syrian family that had migrated to Lebanon. So far, genomic studies have predominantly focused on populations of European ancestry (Fatumo et al., 2022). Jurado-Camacho et al. have reported several novel variants of genes involved in lipid metabolism among the Mexican population. Genetic testing of underrepresented non-European populations can help identify the actual burden of rare dyslipidemias in different genetic backgrounds, and may help guide diagnostic and therapeutic approaches (Al-Waili et al., Jurado-Camacho et al.).

Unlike FCS, multifactorial chylomicronemia syndrome is a complex, often polygenic form of severe hypertriglyceridemia that results from multiple underlying genetic factors coupled with environmental triggers, including medications (Goldberg and Chait, 2020). An example of an interaction between genetic and non-genetic determinants was seen in a normolipidemic patient with a novel apo C-III (*APOC3*) variant who experienced drug-induced hypertriglyceridemia (Iannuzzi et al.).

Lysosomal acid lipase deficiency (LAL-D, also called cholesterol ester storage disease) is an autosomal recessive disorder, caused by biallelic pathogenic variants in the *LIPA* gene encoding lysosomal acid lipase. Besler et al. comprehensively reviewed several aspects of LAL-D, including the role of *LIPA* variants in predicting ASCVD risk from genome-wide association studies and also the cell type-specific role of enhancing LAL activity as a novel treatment strategy of ischemic cardiovascular disease and fatty liver. LAL-D is globally underdiagnosed, partly due to clinical features that may resemble more common dyslipidemias and fatty liver disease, and partly due to lack of access to genetic and enzyme activity testing in clinical practice. Early diagnosis followed by timely management with intravenous enzyme replacement therapy (sebelipase) are achievable by reproducing the Slovenian Universal FH screening strategy which has helped identify patients homozygous for *LIPA* pathogenic variants. LAL-D-positive children have higher liver transaminases (AST and ALT) that clinically differentiate them from FH patients (Sustar et al.).

Several rare dyslipidemias can present with either low LDL cholesterol (hypobetalipoproteinemia) or low HDL cholesterol (hypoalphalipoproteinemia) (Hegele et al., 2020). Disorders characterized by decreased HDL cholesterol, also called familial hypoalphalipoproteinemia, include Tangier disease, an autosomal recessive disorder caused by pathogenic variants in *ABCA1* encoding the ATP binding cassette transporter A1, which is responsible for modulating the flux of cellular cholesterol and phospholipids into the reverse cholesterol transport pathway. Individuals with Tangier disease appear to have a higher incidence of coronary artery disease than normolipidemic subjects, irrespective of gender. Other forms of familial hypoalphalipoproteinemia include those caused by pathogenic variants in *APOA1* encoding apo A-I and *LCAT* encoding lecithin cholesterol acyltransferase (Oram, 2000; Hegele et al., 2020). Alves et al. presented 7 cases with rare dyslipidemias associated with either low LDL or low HDL cholesterol values, who were evaluated with next-generation DNA sequencing. The genetic basis was confirmed in 6/7 patients: one had fisheye disease due to variant *LCAT*, one had hypoalphalipoproteinemia and 5 had either abetalipoproteinemia due to *MTTP* variants or familial hypobetalipoproteinemia (FHBL) due to *APOB* variants. These results stress the important role of genetic testing in the diagnosis of rare dyslipidemias (Alves et al.). Another paper by Molk et al. reports a novel pathogenic *APOB* variant in a child with heterozygous FHBL and non-alcoholic fatty liver disease and comprehensively reviews the existing literature on *APOB* variants causing heterozygous FHBL.

Dysbetalipoproteinemia (also called hyperlipoproteinemia type III, HLP3) is characterized by elevated levels of both triglycerides and cholesterol due to abnormally elevated remnant particles resulting in part from homozygosity for the apo E2 isoform (Bea et al., 2023). This lipid profile is highly atherogenic and predisposes to premature ASCVD. To overcome technical issues in the clinical diagnosis of HLP3, Sampson et al. have described a validated equation for the indirect calculation of cholesterol content of VLDL which is abnormally increased in HLP3.

Cerebrotendinous xanthomatosis (CTX) is a rare lipid storage disease, caused by deficiency of sterol-27-hydroxylase (CYP27). Patients with CTX present with elevated cholesterol, xanthomas, and neurological deterioration that can lead to premature death. Cohen et al. reported detailed metabolic abnormalities and premature atherosclerosis among CTX patients.

The management of rare dyslipidemias is challenging and depends on accurate and timely diagnosis. Genetic testing promises greater accuracy and also insight into pathogenesis (Guo et al., Alves et al.). The discoveries of rare variants in extreme and unexplained phenotypes, with the help of advanced genetic techniques, might help us better understand pathophysiology and lead to the discovery of novel therapeutic targets. Deng et al. describe a proband with sitosterolemia and its successful treatment with ezetimibe. Several promising therapeutic approaches for dyslipidemias are in the pipeline (Merćep et al., 2022). For instance, lomitapide, an inhibitor of microsomal triglyceride transfer protein (MTP) is an important therapeutic option for difficult-to-manage HoFH patients (Kayikcioglu et al.). Interestingly, D’Erasmo et al. have demonstrated the efficacy and safety of lomitapide in autosomal recessive hypercholesterolemia (ARH), an ultra-rare autosomal recessive disorder of LDL metabolism resembling HoFH caused by pathogenic variants in *LDLRAP1* encoding the LDL receptor adaptor protein 1.

In summary, the Research Topic “*Rare dyslipidemias*” has further expanded knowledge on several aspects of these conditions, including their genetic background, phenotypic characteristics, and treatment. Many ethnic groups remain underrepresented in genomic studies. Genetic testing and applicable screening strategies should be more broadly implemented. Collaborative registries are also required to improve health policy for the care of patients with rare dyslipidemias (Hegele et al., 2020).
